# Implementing Smarter Lunchrooms Makeovers in New York state middle schools: an initial process evaluation

**DOI:** 10.1186/s13690-016-0153-9

**Published:** 2016-09-30

**Authors:** Laura N. Thomas, Tisa F. Hill, Alisha Gaines, Jamie S. Dollahite

**Affiliations:** 1 Division of Nutritional Sciences, Cornell University, 342A Martha Van Rensselaer Hall, Ithaca, NY 14853 USA; 2 Division of Nutritional Sciences, Cornell University, 343 Martha Van Rensselaer Hall, Ithaca, NY 14853 USA; 3 Division of Nutritional Sciences, Cornell University, 408 Savage Hall, Ithaca, NY 14853 USA

**Keywords:** Behavioral economics, Process evaluation, RE-AIM, School meals, Childhood obesity, Nutrition

## Abstract

**Background:**

This paper presents design and findings from the process evaluation of a randomized controlled trial (RCT) testing the effectiveness of Smarter Lunchrooms Movement (SLM) interventions to encourage consumption of either fruit, vegetables, or unflavored milk in middle school cafeterias (grades 6–8, typically children ages 10–14 years). Using the RE-AIM (Reach, Effectiveness, Adoption, Implementation, and Maintenance) framework adapted for environmental interventions, the process evaluation monitored fidelity to SLM protocol, determined barriers and facilitators influencing fidelity, and identified the training and support needs of implementers.

**Methods:**

Under research team guidance, community partners (interventionists) assisted school food service staff (providers) with a six week implementation of protocol items in 13 public middle school cafeterias (two milk treatment, three vegetable treatment, four fruit treatment, and four control) in New York State during the 2013–2014 academic year. Process evaluation measures included semi-quantitative measures of implementation and maintenance (lunchroom audits) and qualitative data (environmental assessments and semi-structured interviews with school food service staff). Analyses identified challenges and opportunities for improving intervention delivery.

**Results:**

Approximately 75 % of enrolled students participated in school lunch programs and thus were exposed to the SLM intervention. Findings indicated potential contamination by other nutrition-related activities in the lunchroom and larger school environment may have affected the intervention impact. Modest implementation fidelity scores were observed for intervention treatments. Providers reported treatments were acceptable and feasible, however interventionists confirmed motivation and perceived effectiveness varied among schools. Post-intervention audits revealed limited maintenance of intervention protocols. Strategies to enhance buy-in and communication among providers and increased interventionist support are recommended.

**Conclusions:**

RE-AIM was a valuable framework for this process evaluation. Results highlighted implementation barriers and facilitators, and findings will enhance interpretation of forthcoming outcome data. Results will inform future iterations of the SLM RCT and provide valuable insights for those designing environmental interventions in school cafeterias.

## Background

Childhood obesity remains a global health concern, particularly among industrialized countries such as the United States (US), in which 2014 prevalence among children 2–19 years old was 17 %, with children of racial/ethnic minorities and children of low-income families at increased risk [1, 2]. These data have provided no evidence of a decline in prevalence, and the association between childhood obesity and comorbidities [3], as well as the persistence of obesity into adulthood [4], has been well documented. Additionally, adolescence represents a time of biological and social development during which decreases in overall diet quality and adoption of unhealthful food habits have been observed [5].

Schools have the unique ability to support child health via education, physical activity, and food environments, and have thus been identified as ideal locations for obesity prevention programs [6]. Reviews indicate that school health interventions have had limited success decreasing obesity and improving dietary intake, though randomized controlled trails (RCTs) with nutrition components were promising [7, 8]. The school food environment has received particular attention, given the broad reach of school meal programs and the potential negative impact of food and beverages of minimal nutritional value offered outside of meal programs [9]. Thus common approaches to improve student health have included policy changes to restrict these competitive foods in school settings [10].

Smarter Lunchrooms Movement (SLM) strategies are based on the behavioral science principle of *libertarian paternalism*, which posits that food selection can be influenced by changing behavioral cues without restricting choices [11–13]. SLM pilot studies have indicated that subtle changes to the school lunchroom environment, which make the healthier choices the easier choices for students, may increase selection and consumption of more nutritious foods [12, 14–21]. The present study was the first in a series of RCTs initiated to test the effectiveness of SLM interventions in middle schools (grades 6–8, students typically ages 10–14 years).

In addition to a rigorous experimental design to test effectiveness, it was imperative that this type of intervention be subject to process evaluation. Process evaluations serve multiple functions, including helping to prevent a type III error [22] by monitoring and assuring quality of intervention implementation fidelity, identifying barriers and facilitators to implementation, and assessing the extent to which the intervention is sustained after the study has ended (i.e., maintenance) [23, 24]. Results can facilitate understanding of the internal and external validity of the intervention [25], thus helping interpret intervention results and informing translation of research to practice. The RE-AIM (Reach, Effectiveness, Adoption, Implementation, and Maintenance) framework serves as a useful foundation for designing process evaluation and for reporting salient intervention dimensions, and it was adapted for use in the current study [26–29]. This paper presents the design and findings of the process evaluation conducted for the first of three years of the SLM RCT, during which strategies were tested in isolation to determine individual effectiveness prior to testing in combination in year two.

## Methods

### Intervention design

This RCT was developed in response to a United States Department of Agriculture (USDA) Agriculture and Food Research Initiative request for proposals to target obesity reduction and prevention among middle school age children and was approved by the Cornell University Institutional Review Board (reference number 1203002914A004). Using small, low- or no-cost changes to the school lunchroom, the primary goal was to increase selection and consumption of fruits, vegetables, or unflavored milk offered to students as part of the National School Lunch Program (NSLP). The full list of protocol items can be found in Table 3. Briefly, 15 items were included in the fruit protocol, 13 in the vegetable protocol, and nine in the milk protocol. These items were grouped into three domains: 1) placement and display, which included positioning vegetables after the entrée and offering fruit at multiple points on the service line, 2) creative naming, which entailed posting cards with creative names for fruits, vegetables, and milk on service lines and incorporating names into school menus, and 3) nutrition messaging that included new, rotating nutrition factoid posters in the cafeteria. Fruit and vegetable protocols included an additional “variety” domain, which included offering more than one type of fruit and vegetable daily.

In order to determine the effectiveness of SLM in a variety of settings, the intervention was conducted in a convenience sample of New York State urban and rural middle schools during the 2013–2014 academic year. Eligible schools were public institutions participating in NSLP with dedicated lunch times or serving lines for middle school students. Schools that served other grades concurrently were ineligible because of the inability to collect middle school student data separately.

Researchers partnered with local Cooperative Extension nutrition educators (referred to as *interventionists*) to deliver the intervention in schools, thus schools were recruited from areas in which there were available interventionist staff. A school sampling frame was developed based on the number of students participating in NSLP and balanced representation of urban and rural school districts [30]. Schools were recruited until total NSLP participation across all schools allowed for the minimum number of tray waste observations needed to detect a 5 % change in consumption for each treatment condition, determined using sample size calculations (80 % power) based on previous SLM studies [16, 17, 19, 31]. Schools were randomized to control or intervention groups, for a total of two milk treatment, three vegetable treatment, four fruit treatment, and four control schools. (Schools were balanced by the number of NSLP participating students, resulting in an uneven number of schools in each treatment.) The milk treatment was administered over six weeks in the fall 2013 semester, while fruit and vegetable treatments were administered separately and in different schools during six weeks of the spring 2014 semester.

Interventionists were responsible for recruiting schools, training school food service staff (referred to as *providers*) in the SLM protocol, and conducting weekly contact with providers to answer questions, troubleshoot challenges, and encourage treatment fidelity during the intervention period. Additionally, based on feedback from interventionists and providers in fall 2013, interventionists worked with middle school children to develop creative names used to promote fruits and vegetables on lunchroom signage in spring 2014 [18]. "Ice Cold White Milk" was the pre-assigned promotional name on relevant signage in fall 2013.

### Process evaluation framework, measures, and analyses

The RE-AIM framework was adapted to satisfy the needs of an environmental intervention [26–28]. The definitions and associated process evaluation measures of Reach, Effectiveness, Adoption, Implementation, and Maintenance are described in Table 1. (Effectiveness has been interpreted as potential external influences on the intervention, whereas overall effectiveness of the SLM interventions, measured by student food selection and tray waste, will be reported in a forthcoming paper.) All described process evaluation measures were developed for this study and reviewed by the research team for face validity.

**Table 1:** RE-AIM dimensions and application to process evaluation measures

Dimension	Definition^a^	Process evaluation measures
Reach	Number of people and percentage of the target population affected by the environmental change (and the extent to which the individuals reached are representative and include those most at risk).	Described using proportional reach, i.e., the number and proportion of enrolled students participating in the National School Lunch Program, including the number of low-income^b^ students. Data were retrieved from the New York State Department of Education [29].
Effectiveness	A measure of effects on health behaviors, including positive, negative, and unanticipated consequences.	Described as potential external influences on intervention effectiveness, i.e., contamination or threats to internal validity and aspects of cafeteria environments aligned with protocol prior to implementation, captured by *school environmental assessments* and *lunchroom audits*. Note: Student behavioral outcomes will be reported elsewhere.
Adoption	Number, proportion, and representativeness of settings and intervention agents participating, and the extent to which the settings selected are representative of settings that the target population will use or visit.	Number and characteristics of participating schools. Also described as the number of interventionists and providers trained in Smarter Lunchrooms protocol and their reported preparedness to initiate intervention, detailed in provider training *progression records*, interventionist and provider training *evaluations* and *interviews,* and *contact logs*.
Implementation	Level of adherence to implementation guidelines and the extent to which elements are implemented.	Described as fidelity (including fidelity scores by school service lines) to intervention protocols assessed via *contact logs*, *lunchroom audits* and interventionist and provider *interviews*.
Maintenance	At the setting level, the extent to which change is maintained or new barriers are prevented or reduced.	Described as fidelity scores by school service lines to intervention protocols beyond the intervention end date, assessed via *lunchroom audits* two weeks post-intervention.

^a^Adapted from [26–28]

^b^ Students are classified as low-income if they or their family participate in federal economic assistance programs [29]

Qualitative and semi-quantitative methods were employed to collect process data for the dimensions of the adapted RE-AIM framework: school environmental assessments, school lunchroom audits, provider training records and evaluations, weekly contact logs, and post-intervention semi-structured interviews.

With assistance from school administrators and staff, interventionists completed *school environmental assessment* forms created to document school wellness policies and committees, parent and teacher association activities, nutrition and health education in classrooms, and other relevant health promotion activities in participating schools. Researchers reviewed these assessments for evidence of contamination or co-occurring activities that might impact student selection and consumption of foods promoted through the intervention.

*School lunchroom audits* were conducted in each school before, during, and after the intervention. The mid-intervention audit occurred during week three or four of the intervention to ensure providers had enough time to enact the protocol comfortably, avoiding days in which tray waste was collected to minimize the potential of compounded observer effect. Audits were performed by the research team and included field notes, a comparison of the intervention protocol items with actual lunchroom and service line set-ups, diagrams of each line set-up, and photographs of each line. To generate a semi-quantitative measure of implementation fidelity, each service line received a score for each intervention protocol item: 2 for full compliance, 1 for partial compliance, and 0 for non-compliance. Scores were calculated for all service lines in participating schools. Line scores were averaged within schools, and averages across schools in urban and rural areas were calculated. Averages across all schools were calculated to provide an idea of intervention compliance.

The research team conducted interventionist training. The two-day trainings, designed using Principles of Adult Learning Theory [32] to accommodate multiple learning styles, included an introduction to behavioral economics, project overview and logistics, and a walkthrough of the expected provider training. Interventionists completed *evaluation* forms following these trainings in which understanding and preparedness were assessed with 12 items using a 5-point Likert scale (strongly disagree – strongly agree). Items included statements such as:

“…I have a better understanding of the study design, including the intervention, process evaluation, and outcome evaluation.”

“…I understand the differences between activities in intervention and control schools.”

“…I feel prepared to deliver training to intervention school staff.”

Item responses were converted to scores and averaged within and across all schools. A total mean across all items was also calculated. Open-ended questions were used to solicit opinions regarding valuable aspects of training and suggestions for improvement. These results were reviewed and summarized for all interventionists.

Interventionists delivered treatment-specific (milk, fruit, or vegetable), one-hour trainings to providers. Control school providers participated in an unrelated kitchen and food safety training created by the National Foodservice Management Institute [33]. Trainings were based on Adult Learning Theory [32] and designed for an audience with a low literacy level in response to provider needs. Specific learning objectives for providers in treatment schools were 1) to develop a working knowledge of SLM rationale, 2) to gain information related to the SLM research project description, purpose, team and timeline, 3) to develop a firm understanding of the specific change to be made in their school’s lunchroom, and 4) to practice using implementation tools. *Training records* were completed by interventionists and used to document training delivery. Records were reviewed for indicators that the training had been delivered as intended, and any modifications were noted. Providers completed a training *evaluation* that consisted of ten items assessing understanding and preparedness to implement the intervention using a 5-point Likert scale (strongly disagree – strongly agree), as well as open-ended questions to further solicit training feedback.

Likert scale items included statements such as:

“…I know what changes need to be made in my school lunchroom.”

“…I am comfortable with Smarter Lunchrooms tools (sings, planograms, etc.).”

“…I am prepared to carry out my role in the Smarter Lunchrooms project.”

As with interventionist evaluations, statement responses were averaged and a total mean calculated. Responses to open-ended questions were reviewed and summarized for all providers.

Weekly *contact logs* were completed by the interventionists to record providers’ experiences with implementation, including any challenges or concerns. Logs were also reviewed for evidence of adoption/implementation fidelity. Additionally, members of the research team conducted post-intervention *semi-structured interviews* with a sample of interventionists and providers regarding their experiences implementing SLM in their schools in order to understand barriers and facilitators to implementation, determine the strengths and weaknesses of trainings, and clarify which elements of the intervention were maintained. All 16 interventionists and at least one provider from each of the 12 participating treatment schools (typically a manager with knowledge of day-to-day activities) were invited to participate in post-intervention interviews.

Interventionists were asked about recruitment of schools, training they received, training they delivered to providers, and the help and support they received from the research team. Providers identified challenges with implementation, provided feedback on their training, delineated support they received from interventionists, and described any observed intervention outcomes. Interviewees were also asked if there was other information they would like to discuss. Interviews were recorded and transcribed verbatim. Researchers used ATLAS.ti (version 7, 2012, ATLAS.ti Scientific Software Development GmbH, Berlin, Germany) qualitative data management software to code commonalities as well as discrepancies in reported successes and challenges by treatment and across sites.

## Results

### Reach

Characteristics of the 13 participating schools are further described in Adoption below. Approximately 2,100 (75 %) 6^th^-8^th^ grade students enrolled in treatment schools participated in NSLP throughout the academic year and thus were exposed to the interventions (Table 2), including about 455 students from low-income families.

**Table 2:** Demographic characteristics of participating schools and enrolled students, Smarter Lunchrooms Makeovers RCT, New York State, 2013–2014

	Fall 2013			Spring 2014									
School treatment	Milk	Milk	Control	Vegetable	Vegetable	Vegetable	Fruit	Fruit	Fruit	Fruit	Control	Control	Control
Designation	Urban	Rural	Rural	Urban	Rural	Rural	Urban	Urban	Rural	Rural	Urban	Urban	Rural
Lines represented (n lines)	3	4	2	4	6	1	2	3	2	2	3	2	2
Total enrollment grades 6–8 (n students)	192	755	892	369	159	295	151	233	327	315	91	193	386
Average NSLP participation (% students)	95	72	57	92	60	50	95	95^a^	59	72	100	84	59
Reach^b^ (n students)	183	542	508	338	96	147	144	223	192	227	91	163	226
Low-income students (% students)	82	50	51	71	64	38	85	91	57	53	91	85	49
Race/Ethnicity (% students)													
Black	92	7	12	23	6	6	93	18	1	2	33	85	4
White	2	77	62	57	90	89	2	6	92	97	20	5	90
Hispanic	3	9	16	3	3	3	3	73	4	<1	7	5	4
Other	3	7	10	17	1	2	2	3	3	<1	40	5	2
Gender (% students)													
Female	54	49	50	47	46	50	45	50	47	46	56	55	47
Male	46	51	50	53	54	50	55	50	53	54	44	45	53

^a^Missing data. Calculated as the average participation of other schools within the same district

^b^Calculated as enrollment x NSLP participation

Schools from the urban district provided free meals to all enrolled students through the Community Eligibility Provision (CEP) [34], eligibility for which is determined based on the percentage of students from low-income families. In these schools, >95 % of students participated in NSLP. No participating rural schools qualified for CEP, thus more urban students (95 %) were reached with the intervention compared with rural students (65 %).

### Effectiveness

Process data highlighted several possible sources of contamination within participating schools that may have affected student food selection and tray waste. School environmental assessments revealed that all treatment schools had established wellness policies that varied in content and active contribution to school food and nutrition education environments, and urban treatment schools operated under the same district-wide policy. Policies most often addressed meeting requirements of the federally established NSLP and integrating nutrition education in the classroom. Curricula in all schools incorporated nutrition education components in health or family and consumer sciences courses, which most often included Supplemental Nutrition Assistance Program Education (SNAP-Ed) fruit and vegetable activities from the Cooking Matters curriculum [35].

Schools were asked to delay introduction of new extracurricular food and nutrition education programming if possible; one urban milk treatment school suspended a weekly nutrition program for the duration of the intervention. However, direct nutrition education programming was occurring in two rural treatment schools, one fruit and one vegetable, during the intervention period. Programming included USDA MyPlate and Supertracker meal and activity planning [36], the Choose Health: Food, Fun, and Fitness healthy eating and active living curriculum [37], and National Nutrition Month activities [38] in the spring semester.

Pre-intervention school lunchroom audits identified newly adopted cafeteria practices developed in response to changes in the NSLP regulations [39]. SLM intervention protocol dictated that treatment schools increase fruit and vegetable selections to at least two varieties per line. However, all participating schools were already offering a minimum of two fruits and two vegetables in response to recent NSLP regulations, rendering this particular component insensitive to change. There was no apparent impact of NSLP changes on the milk treatment. In addition to these changes, audits also revealed that students in urban schools (CEP schools) could select from either one hot or one cold lunch daily, with no competitive foods available, thereby minimizing student choice and differences in food selected by students. Students in rural schools were exposed to larger numbers of competitive food options.

### Adoption

#### School-level adoption

Of the 17 schools invited to participate, 13 (nine treatment, four control) across eight school districts in five counties (one urban, four rural) participated in this RCT. See Table 2 for further school demographic characteristics. Adoption occurred at the food service director level with approval of school administration. Two school food service directors representing four schools in two rural counties were unwilling to participate in the intervention, citing negative experiences with previous involvement in research studies.

#### Interventionist- and provider-level adoption

All 16 interventionists received the training conducted by researchers and completed the training evaluation. Mean rating across items was 4.5 out of 5 (range: 4.0 - 4.9), indicating interventionists agreed or agreed strongly that they understood the project purpose and their roles and felt equipped to initiate study responsibilities. Data from open-ended evaluation questions and post-intervention interviews with 14 interventionists (88 % response) revealed that they found the trainings detailed and well organized. They enjoyed the hands-on nature of the activities and liked the training materials.

However, due to production and shipping delays, interventionists did not have access to the complete set of intervention materials needed to train the providers, which negatively impacted their ability to effectively train the providers for successful implementation of SLM in a timely manner. Interventionists suggested that the full complement of these materials be available at future trainings. Interventionists in the urban locations also requested the development of a module with guidance on how to increase buy-in from reluctant or less enthusiastic providers.

Urban Interventionist: “I guess one thing would be maybe some guidance regarding if… they agree to buy in at the food service director level, but at the front-line level, in this case food service staff, they're not really on board with it. So, some direction there.”

Interventionists reported they were comfortable delivering the training to providers, as it was designed in accordance to their standard programming. Training records indicated that all components of provider trainings were delivered in all treatment schools; however, both urban and rural interventionists reported enhancing the trainings to be more responsive to provider learning styles and to increase provider buy-in. For example, urban interventionists reported offering fruit and vegetable snacks during provider trainings as both an incentive to participate as well as a teaching tool.

Training records also indicated that interventionists in the urban schools encountered unexpected time constraints and typically had only 20–25 min to deliver one-hour provider trainings. Urban interventionists indicated that trainings typically took place during providers’ lunch breaks and that providers often left before the end of the training to continue setting up the lunch line. As a result, interventionists believed that the training was disruptive to providers and may have generated a negative attitude towards the intervention from the onset. One urban interventionist noted, “It’s already coming in a negative manner. You know? ‘Oh, you’re interrupting our lunch.’” Both urban and rural interventionists spoke of the need to include more information in the training for providers about how SLM strategies could positively impact the school food service staff, students, and the food service budget.

Rural interventionist: “If there’s more information about how this benefits the kids ultimately, or benefits, you know, the cafeteria staff… If it’s less things going in the garbage, it could help your bottom line… Whatever we can bring back to show them the benefits would be good.”

Sixty-one of 63 providers attending trainings completed training evaluations (97 % response), and eight of 12 providers (67 % response) in manager positions participated in post-intervention interviews. Interview participation varied based on response to this invitation and willingness to participate, difficulty scheduling interviews, and inclement weather and road conditions that prohibited both travel and whether or not schools were open/staff were available. Training evaluation responses indicated mean understanding and preparedness was 4.1 out of 5 (range: 3.0 - 4.9). Providers were less likely to report that they believed the intervention would benefit students or that they were excited to begin the intervention. Providers were most likely to indicate that they were prepared to carry out their role in this project and that they knew who to contact with questions. Interview data also indicated that providers believed the training was effective, and providers offered few suggestions for training improvements.

### Implementation

Table 3 includes average fidelity scores for the protocol items pre-, during, and post-intervention for urban and rural treatment schools’ service lines. For each treatment domain, scores were averaged across lines, across schools in urban and rural groups, and finally across all schools.

**Table 3:** Protocol description and mean implementation fidelity scores^a^ for treatment school lines, Smarter Lunchroom Makeovers RCT, New York State, 2013–2014

Protocol category	Protocol items	Pre-intervention			During intervention			Post-intervention		
Unflavoured Milk Treatment		Urban (1 school, 3 lines)	Rural (1 school, 4 lines)	Total (2 schools, 7 lines)	Urban (1 school, 3 lines)	Rural (1 school, 4 lines)	Total (2 schools, 7 lines)	Urban (1 school, 3 lines)	Rural (1 school, 4 lines)	Total (2 schools, 7 lines)
Placement & Display	1. Unflavored milk displayed in front of sugar added beverages 2. Unflavored milk makes up 1/3 of all visible milk 3. Unflavored milk within students’ reach	0.4	0.9	0.7	1.6	0.8	1.2	-	-	-
Creative Naming	4. Display “Ice Cold White Milk” sign near milk cooler 5. “Ice Cold White Milk” written on daily menu board 6. “Ice Cold White Milk” written on weekly menu	0.0	0.0	0.0	0.7	0.3	0.5	-	-	-
Nutrition Messaging	7. Additional signage 8. Display dry erase board 9. Include nutrition messaging on dry erase board	0.0	0.0	0.0	1.7	0.5	1.1	-	-	-
Column mean		0.1	0.3	0.2	1.3	0.6	0.9	-	-	-
Vegetable Treatment		Urban (1 school, 4 lines)	Rural (2 schools, 7 lines)	Total (3 schools, 11 lines)	Urban (1 school, 4 lines)	Rural (2 schools, 7 lines)	Total (3 schools, 11 lines)	Urban (1 school, 4 lines)	Rural (2 schools, 7 lines)	Total (3 schools, 11 lines)
Placement & Display	1. Hot vegetable placed right after hot entrée 2. Vegetables displayed in at least 2 locations on a given line^b^ 3. Use cups provided by intervention to hold raw vegetables/salads 4. All vegetable bowls/cups easy to see on line^b^	1.0	0.9	1.0	1.3	1.1	1.1	1.0	1.2	1.1
Creative Naming	5. Cards with creative vegetable names next to all vegetable displays and easy to see 6. Creative vegetable names appear on monthly menu 7. Creative vegetable names appear on daily menu board 8. Small dry erase boards have hand-written, colorful creative vegetable names 9. Small dry erase boards easy to see	0.0	0.0	0.0	1.2	0.3	0.6	0.0	0.2	0.2
Nutrition Messaging	10. Large dry erase boards with vegetable factoids easy to see 11. New vegetable factoid on board each week 12. Small dry erase boards with vegetable messages easy to see	0.0	0.0	0.0	1.0	0.8	0.9	1.3	0.2	0.6
Variety	13. At least two kinds of vegetables on line^b^	2.0	1.4	1.6	2.0	0.9	1.3	2.0	1.3	1.5
Column mean		0.8	0.6	0.6	1.4	0.8	1.0	1.1	0.7	0.8
Fruit Treatment		Urban (2 schools, 5 lines)	Rural (2 schools, 4 lines)	Total (4 schools, 9 lines)	Urban (2 schools, 5 lines)	Rural (2 schools, 4 lines)	Total (4 schools, 9 lines)	Urban (2 schools, 5 lines)	Rural (2 schools, 4 lines)	Total (4 schools, 9 lines)
Placement & Display	1. Fruit placed first on line 2. Fruit displayed in at least 2 locations on line 3. Use cups provided by intervention to hold fruit 4. All fruit bowls/cups easy to see on line^b^ 5. Fruit bowls/cups are within reach^b^ 6. Separate fruit primer bowl visible	0.9	0.9	0.9	1.6	1.4	1.5	0.7	1.3	1.0
Creative Naming	7. Cards with creative fruit names next to all fruit displays and easy to see 8. Creative fruit names appear on monthly menu 9. Creative fruit names appear on daily menu board 10. Small dry erase boards have hand written, colorful creative fruit names 11. Small dry erase boards easy to see	0.0	0.0	0.0	0.3	0.9	0.6	0.2	0.1	0.2
Nutrition messaging	12. Large dry erase boards with fruit factoids easy to see 13. New fruit factoid on board each week 14. Small dry erase boards with fruit messages easy to see	0.0	0.0	0.0	1.9	1.4	1..7	1.0	0.0	0.5
Variety	15. At least two kinds of fruit on line^b^	2.0	2.0	2.0	2.0	2.0	2.0	2.0	2.0	2.0
Column mean		0.7	0.7	0.7	1.5	1.4	1.4	1.0	0.8	0.9

^a^2 points for full complicance, 1 point for partial compliance, 0 points for non-compliance

^b^Protocol elements already in place prior to study

#### Milk treatment

Overall fidelity across both schools was moderate (0.9/2 points, or 45 % compliance); however, the urban school demonstrated higher overall compliance during the intervention period (65 %) compared with the rural school (30 %). Fidelity was most limited for use of creative naming. Specifically, both schools displayed signage next to milk coolers, while neither added creative names to daily or weekly menus. Placement and display items were implemented most consistently, though because of some pre-intervention adherence within this category, the largest increase in compliance from pre- to during intervention was seen for nutrition messaging. Urban providers implemented both the placement and display and nutrition messaging protocol items with the highest degree of compliance and with substantial increases from baseline to intervention. Increased variety of beverages (seltzer water and other sugar-sweetened beverages) and number of display cases available in the rural school made it impossible for providers to offer unflavored milk in all areas where beverages were available to students, effectively decreasing fidelity scores.

#### Vegetable and fruit treatments

Total fidelity to vegetable protocol items across all three treatment schools during the intervention was approximately 50 %, and there were notable differences among overall compliance in rural (40 %) and urban schools (70 %). Fidelity was highest among variety protocol items in urban and rural schools, though compliance was skewed by 100 % baseline adherence in urban areas. Messaging was the least implemented category across all lines, followed by creative naming. Despite modest fidelity scores, the largest increases in compliance were observed for creative naming in both urban and rural areas, as well as messaging in rural schools.

Total fruit fidelity during the intervention (70 %) was higher than total vegetable fidelity (50 %), with similar compliance among urban and rural schools (75 and 70 %, respectively). Unlike with vegetable variety, both urban and rural schools were in 100 % compliance with fruit variety prior to and during the intervention. Excluding variety, fruit messaging items were implemented with the highest fidelity across all lines and accounted for the largest increases in compliance from baseline. Fidelity was lowest for fruit creative naming protocol items, though rural lines demonstrated higher adherence than urban lines. For both fruit and vegetable treatments, the vast majority of schools failed to add creative fruit or vegetable names to their daily and monthly menus, keeping fidelity scores low.

Additionally, urban and rural school lines exhibited contrasting fidelity to creative naming for the fruit and vegetable interventions. In urban schools, fidelity to vegetable creative naming was higher than similar protocol items for fruit intervention, as the wide variety of fruit offerings created challenges both in matching daily options with the creative names and finding room on the sneeze guard to post all of the fruit signage needed at a given time. While rural schools complied with fruit creative naming at a higher level than similar protocol items for the vegetable intervention, the reasons for this difference were unclear.

Fidelity to placement and display protocol items for fruit compared with vegetables during the intervention was largely attributable to consistent use of primer bowls provided, which increased visibility of whole fruit and was unique to the fruit intervention, and to more consistent use of attractive serving cups in fruit treatment schools compared with vegetable treatment schools.

Data from lunchroom audits suggested that the vegetable treatment received a much lower dose of the intervention than the fruit treatment, even when implementation fidelity was high, due to the relative proportion of fruit offered in lunchrooms as compared to vegetables. Specifically, in any given school there was usually a selection of several fruits available, while there was typically only one hot and one cold vegetable offered each day. Fruit treatment school lines were often saturated with creative fruit name signage, while vegetable signage was less obvious due to limited varieties available.

#### Implementation barriers and facilitators

Many providers liked the materials provided by the intervention and the initial effect on the students. In an urban school, “When the children saw the bowls… they were very excited… when they started grabbing the salad they said ‘Oh!’ Their eyes got big. Their face lit up and they were passing over [to] pick a salad. All the other kids would take one.” A rural provider noted, “I mean, you know they just read [creative name sign], you always had that little giggle or whatever.” Providers also enjoyed the names that the students generated for menu items. “I mean, we had some good comments. You know, they thought it was going to be cute and stuff and nice names that [the students] had chosen.”

However, providers explained that changing materials (i.e., nutrition messaging on the dry erase boards and putting the creative name signs on the line) was very time consuming given time and space constraints in their cafeterias.

Rural provider: “With budgetary cuts, I have a skeleton crew, and you know that five, ten minutes it takes to write that poster up, you know, once a week, especially on a Monday, that’s just very time consuming. Mondays are the worst prep day, I mean, you’ve got to get everything set up for the week.”

In both urban and rural schools, some providers questioned intervention effectiveness and/or expressed negative attitudes related to the perceived ineffectiveness of their efforts. An urban provider believed “…at this particular school, it didn’t impress the children at all. You know, they could care less whether you had it there or not… Some of them didn’t even look at the signs and, and paid no attention to the signs.” Another urban provider said, “I think it’s a complete waste of time.” One rural provider questioned sustained impact, stating, “After the four-week cycle had gone through and we had started again, I didn’t really hear as many comments about it.”

The need for provider buy-in was highlighted by contact logs from schools receiving fruit or vegetable interventions that revealed some urban schools had low levels of implementation fidelity in the first few weeks of the intervention. This was resolved when the school district’s food service director intervened to communicate that intervention participation was mandatory. In the urban county, the whole school district was recruited via the food service director, whereas rural providers opted in on a school-by-school basis, indicative of differing management structures which may have impacted staff motivation.

Additionally, there was a strong sense among providers that they did not have a lot of input in the execution of the intervention, as exemplified by a rural provider: “But based on my staff observations, they kind of felt left out of it. They were just, you know, everybody came in and just told them what they were going to do, and they just kind of did it.”

During post-intervention interviews, both interventionists and providers mentioned the need to generate excitement about the project and potentially offer incentives for providers.

Urban interventionist: “We can create more excitement too, like… ‘This is the tool kit. This is all the stuff you’re gonna have, and you can keep them.’ Some type of incentive and some type of challenge. Well the schools that do… the best job, you know, they all [get taken] out for lunch or have a big… celebration… they can get movie tickets, you know, all different little things…to motivate them.”

### Maintenance

Barriers and facilitators to intervention implementation likely impacted maintenance of treatments as well. Table 3 indicates the extent to which the schools were complying with the fruit and vegetable intervention about two weeks after the end of the intervention period, i.e. in the maintenance period. Due to winter weather travel conditions, maintenance data were not collected for the milk treatment.

Fidelity to placement and display protocol items for both fruit and vegetable treatments returned to baseline levels, though there was modest maintenance in rural schools. Though generally not maintained at intervention levels, nutrition messaging components of fruit and vegetable treatments were typically maintained above baseline. Notable exceptions include continued increase of vegetable messaging in urban schools and abandonment of fruit messaging in rural schools.

Despite noted barriers, lunchroom audits revealed use of creative fruit names were still being employed post-intervention, and while rural providers also maintained some naming for vegetables, urban providers did not. Post-intervention interviews with providers suggested that providers continued to use the signage for the fruits and vegetables. “I mean, the signs do look cute, so we do still use them,” said one urban provider, and a rural provider stated, “We’re still putting the signs up for the vegetables, and we still, I still left the names on the menu and stuff.”

## Discussion

In light of the prevalence of childhood obesity and schools’ unique position to effect changes in student diet and body weight [6], school health interventions are increasingly common, though have been marginally successful [7, 8]. SLM interventions have been shown to positively impact student consumption in pilot studies [12, 14–21] and are of particular interest in middle schools, given that students are experiencing critical physical and psychosocial development [5]. The effectiveness of SLM protocol items on increasing student selection and consumption of fruit, vegetables, or unflavoured milk was therefore tested using a rigorous experimental design with process evaluation based on an adapted RE-AIM framework [26–28].

### Reach

Approximately 75 % of enrolled students were exposed to SLM interventions throughout the 2013–2014 school year. This intervention coincided with the introduction of CEP [34], or universal free lunch. Thus via CEP, reach was effectively expanded in urban schools due to high levels of NSLP participation (coinciding with increased exposure among higher proportions of students from low-income families). Such national policies provide increased opportunities for interventions to complement and augment health impacts, particularly among youth at risk for obesity.

In this study, all students entering the cafeterias could have been exposed to aspects of the intervention, even if not participating in school meal programs, and any intervention-specific changes that were maintained will continue to reach students in subsequent years. Though this likely increased the number and representativeness of students reached, spillover effects were not assessed in the present study.

It is important to note that reach is defined more broadly within environmental interventions, thereby warranting multiple indicators of this dimension [27, 40]. Sweet et al. [40] included several reach categories (intended, direct, and indirect) in their evaluation of multisector partnerships, a methodological consideration for future iterations of this study. Glasgow et al. [41] advocate for evaluation of the combined impact of reach and effectiveness in order to yield integrated data that could more thoroughly inform SLM intervention design and future school policy decisions.

### Effectiveness

Forthcoming outcome results will provide indicators of intervention effectiveness, though because of the dynamic, multi-dimensional school environment, an array of confounding factors is not uncommon [42] and should be assessed before drawing conclusions regarding changes in student food consumption as a direct effect of SLM [43]. Findings indicated the potential for contamination in the larger school environment (e.g., nutrition education) that may have supported positive food choices and thereby diminished the apparent impact of the SLM intervention. Additionally, cafeteria policies and practices related to food availability may have had a range of effects on study outcomes. Increased fruit and vegetable variety in response to NSLP regulation changes [39] had the potential to positively impact student diet, but changes introduced a ceiling effect that should be considered in analyses of outcome data and design of future study protocols.

Conversely, the presence of competitive foods in rural schools may have negatively impacted student intake, and the full food environment will be considered when analyzing the impact of SLM protocol items designed to work within unrestricted food environments. In a systematic review, Chriqui et al. [10] reported mixed effects of competitive food policies on overall student consumption and body mass index and concluded that more and rigorous research is needed to truly understand effect of these policies. SLM pilot work has suggested that student selection and consumption can be improved without restrictions beyond NSLP regulations [12, 14–21], and outcome results of this RCT strengthened by process evaluation data will provide further evidence regarding the effectiveness of such approaches.

### Adoption

A high percentage of invited schools (76 %) agreed to participate in the current study. Adoption was higher than in other studies which have involved school education departments (i.e., teacher involvement or curriculum changes) [42, 44]. Because the NSLP falls under the jurisdiction of USDA, participation was also likely enhanced due to the national recognition of SLM as a USDA-approved program.

In this study, adoption was also described using training results. Research has suggested that well-trained interventionists and providers demonstrate enhanced ability to deliver the intervention, important for treatment fidelity [45]. The high response rates for both interventionist and provider training evaluations and post-intervention interviews lend confidence in the representativeness of data presented. Interventionists indicated high levels of satisfaction with trainings they received. Evaluations suggested that providers felt the training effectively prepared them to execute the intervention and that they did not find the intervention to be too complicated. Interventionists reinforced the need to have the full complement of materials available at the training to both enhance the training experience and potentially impact intervention outcomes by increasing provider motivation and adoption. Intervention design considerations that consider ease and efficiency of delivery have been shown to enhance adoption in other settings as well [24].

However, data from provider training progression records, provider training evaluations, and interviews highlighted the need to generate provider buy-in. In response, the research team will develop a training module informed by these results and fidelity literature [45] which will equip interventionists to emphasize relevancy and benefits of SLM work, such as the prestige of participation in a USDA-funded study, potential reduction of tray waste and enhanced bottom lines, and facilitation of healthful student food choices. Further, to address the issues of time constraints and minimize interruptions, future provider trainings should be scheduled to coincide with in-service days. Incentives will also be considered.

### Implementation and maintenance

School lunchroom audits revealed fruit and vegetable variety protocol items consistently received the highest fidelity scores pre-, during, and post-intervention in both rural and urban schools. However, compared with fruit, vegetable variety fidelity was lower in rural schools due to the limited variety of vegetables available daily. Overall, both urban and rural compliance with each fruit treatment category was higher than observed for vegetables, with the exception of creative naming in urban lines. Another SLM study conducted in Ohio schools found mean implementation scores for fruit practices were higher than scores for vegetable practices [46].

Following variety, placement and display protocol items for fruits, vegetables, and milk were the most consistently implemented, though many were in place pre-intervention and gains observed during the intervention period were lost post-intervention when fidelity scores generally returned to baseline. These results may point to provider comfort with protocol items that reflected their pre-intervention routines, while naming and messaging components were additions to provider routines. Though naming and messaging items accounted for the largest increases in fidelity scores from baseline, fidelity to these components was generally lower than variety and placement/display items, and the reason most commonly cited by providers for inadequate implementation was a lack of time.

Implementation and maintenance fidelity scores may have been an indirect reflection of provider attitudes. Rural providers had more neutral or positive attitudes towards the intervention, whereas the urban providers tended to be more negative, contributing to food service director intervention that increased fidelity. These urban schools were recruited via the district-wide food service director, thus each school’s providers had no say in participation. Rural districts encompassed fewer middle schools with more closely connected food service administration. Schools opted in on a school-by-school basis and may have therefore had different or more widespread motivation to participate compared with urban schools. These results highlight the need to improve provider engagement and buy-in at training and throughout the intervention. Phase two of this RCT will consider provider motivation and reciprocity, as well as the complexity and compatibility of the intervention with specific school settings [28, 45].

### Strengths

This is the first paper to evaluate the process of implementing SLM in schools. Design strengths include observational data, as opposed to self-report, and the use of a mediating staff group (interventionists) that communicated, addressed, and provided timely support for concerns. These results will be used to inform future iterations of the SLM RCT. Beyond this study, results will assist the translation of research to practice in school cafeterias across the US doing SLM work. Additionally, authors have applied the increasingly popular RE-AIM framework designed to facilitate the translation of research to practice [26–28, 41], and dissemination of framework adaptations such as RE-AIM for environmentally focused interventions is timely.

### Limitations

Limitations of the study included use of a convenience sample; selection bias may have been introduced. A limitation related to elements of the process evaluation was the lack of a measure of preparedness to deliver the intervention among the interventionists, which should be added in future iterations of SLM work so that any necessary confidence- and skill-building can be incorporated in subsequent training or intervention communication. Further testing of process evaluation measures is also warranted.

Finally, fidelity scores were calculated from single observation lunchroom audits conducted during the intervention. While triangulation of data from all process measures provided a means of interpreting data, increasing the frequency of fidelity measurements could provide a more accurate assessment of school adherence to SLM. Also, maintenance audits were conducted shortly after the end of the intervention to account for school holidays, and milk maintenance data was not collected. Maintenance is typically defined as a longer term dimension [47, 48], which would provide a more thorough evaluation of maintenance.

## Conclusions

The RE-AIM framework served as a useful foundation upon which to build the process evaluation of a SLM environmental school lunchroom intervention [25–27]. Process evaluation data were used to monitor protocol implementation fidelity, understand barriers and facilitators to adoption, and to determine whether protocol items were maintained after the study ended. Results indicated the intervention was generally feasible, though it could be enhanced by consideration of school-level policies in place and staff engagement.

SLM is an approved intervention in the USDA SNAP-Ed Obesity Prevention Toolkit for States [49], which means that many SNAP-Ed nutrition providers are interested in incorporating SLM in their programs. However, if the intervention is not delivered as intended, the outcomes will not reflect the original research [12, 14–19], limiting intervention ability to effect childhood obesity. Given the extensive reach of school meals programs, research and program implementers need to work more closely with providers in the schools to address and reduce school-specific barriers to implementation.

Lastly, this paper provides important information regarding the training of interventionists and providers to deliver SLM in both urban and rural school settings. Findings from this work may be useful for researchers and practitioners interested in implementing similar environmental changes in school settings and are particularly relevant as the focus of community-based organizations are shifting focus toward sustainable environmental changes for obesity prevention. Results will inform future study implementation, can be used to advise SLM projects across the nation, and will guide the development of a training model that can be disseminated nationally.

## Abbreviations

CEP: Community eligibility provision
NSLP: National School Lunch Program
RCT: Randomized controlled trial
RE-AIM: Proper name of the evaluation framework with the following domains: reach, effectiveness, adoption, implementation, and maintenance
SLM: Smarter Lunchrooms Movement
SNAP-Ed: Supplemental Nutrition Assistance Program Education
USDA: United States Department of Agriculture
